# Changes in multi-segment foot biomechanics with a heat-mouldable semi-custom foot orthotic device

**DOI:** 10.1186/1757-1146-4-18

**Published:** 2011-06-21

**Authors:** Reed Ferber, Brittany Benson

**Affiliations:** 1 Faculty of Kinesiology, University of Calgary, Calgary, AB, Canada; 2 Faculty of Nursing, University of Calgary, Calgary, AB, Canada; 3 Schulich School of Engineering, University of Calgary, Calgary, AB, Canada

## Abstract

**Background:**

Semi-custom foot orthoses (SCO) are thought to be a cost-effective alternative to custom-made devices. However, previous biomechanical research involving either custom or SCO has only focused on rearfoot biomechanics. The purpose of this study was therefore to determine changes in multi-segment foot biomechanics during shod walking with and without an SCO. We chose to investigate an SCO device that incorporates a heat-moulding process, to further understand if the moulding process would significantly alter rearfoot, midfoot, or shank kinematics as compared to a no-orthotic condition. We hypothesized the SCO, whether moulded or non-moulded, would reduce peak rearfoot eversion, tibial internal rotation, arch deformation, and plantar fascia strain as compared to the no-orthoses condition.

**Methods:**

Twenty participants had retroreflective markers placed on the right limb to represent forefoot, midfoot, rearfoot and shank segments. 3D kinematics were recorded using an 8-camera motion capture system while participants walked on a treadmill.

**Results:**

Plantar fascia strain was reduced by 34% when participants walked in either the moulded or non-moulded SCO condition compared to no-orthoses. However, there were no significant differences in peak rearfoot eversion, tibial internal rotation, or medial longitudinal arch angles between any conditions.

**Conclusions:**

A semi-custom moulded orthotic does not control rearfoot, shank, or arch deformation but does, however, reduce plantar fascia strain compared to walking without an orthoses. Heat-moulding the orthotic device does not have a measurable effect on any biomechanical variables compared to the non-moulded condition. These data may, in part, help explain the clinical efficacy of orthotic devices.

## Background

Foot orthoses have been shown to be efficacious for the treatment of running-related musculoskeletal injuries [1,2]. In terms of pain relief, success rates of between 70% and 90% have been cited [1,3-5]. It has also been reported that 53% to 83% of patients continue to wear their orthotic devices even after their symptoms have been resolved [6,7]. Thus, these results strongly suggest that foot orthoses are effective in the treatment of overuse injury. However, while the clinical efficacy of orthotic devices is widely documented, the mechanism behind that success is not well understood [1,3-5,8].

Several studies have investigated changes in lower extremity kinematics while running and walking in orthoses. Unfortunately, the vast majority of these studies have focused on rearfoot mechanics producing varying results. While some studies have shown no effect of orthoses on rearfoot mechanics [9-11], most studies have shown that foot orthoses control some aspect of rearfoot kinematics, such as peak eversion, eversion velocity or eversion excursion [8,12-15]. However, no study has investigated whether orthoses, either custom-made or semi-custom in design, alter midfoot kinematics.

Most orthotic devices have some type of arch support that either conforms to the shape of the medial longitudinal arch or functions to control arch deformation [8]. Thus, it has been hypothesized that orthoses may function to minimize strain to the plantar fascia tissue through arch control [16,17]. Moreover, Williams et al. [15] suggested that orthotic devices could influence midfoot kinematics possibly by minimizing arch motion during running. Since more biomechanical research is needed to understand the mechanics underpinning the clinical efficacy of orthoses, the purpose of this study was to determine changes in multi-segment foot biomechanics during shod walking with and without an orthotic device. We chose to investigate a semi-custom orthotic device that incorporates a heat-moulding process, to further understand if the moulding process would significantly alter rearfoot or midfoot kinematics and plantar fascia strain as compared to a no-orthotic condition. We hypothesized the semi-custom device, whether moulded or non-moulded, would reduce peak rearfoot eversion, peak tibial internal rotation, medial longitudinal arch angle, and plantar fascia strain, compared to the no-orthoses condition. We also hypothesized that the non-moulded orthotic condition would serve to minimize arch deformation, and thus minimize plantar fascia strain and medial longitudinal arch angle, more so as compared to the moulded condition.

## Methods

### Participants

Twenty healthy individuals (9 males, 11 females: age = 24.6 ± 4.9 years, height = 176.5 ± 8.6 cm, mass = 75.9 ± 11.7 kg) volunteered to participate in the study. All participants were currently free from lower extremity injury, had no prior history of surgery, and were familiar with treadmill walking. The institutional ethics board approved the study, and written informed consent was obtained from all participants.

### Orthotic device

**Figure 1:**
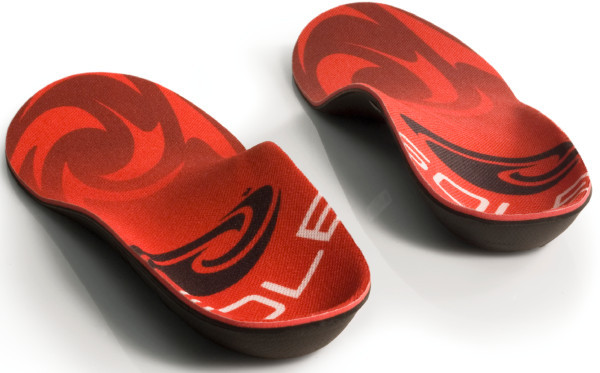
**The semi-custom mouldable Softec Response orthotic used in the current study**.

An over-the-counter, semi-custom orthotic device (Softec Response orthotic; SOLE Inc., Calgary, Canada) was used in the present study (Figure 1). The footbeds were made from foam with a hardness of Asker (C): 70-75. All manufacturer instructions were followed during the moulding process such that the orthotic device was placed in a 90°C pre-heated oven until the "Opti-therm" indicator turned black, or three minutes had passed, whichever came first. The orthotic devices were then immediately placed inside both shoes and the participant placed their feet overtop, laced up the shoes, and stood upright for 2 minutes for the moulding process to complete. For all participants, the right limb was chosen for analysis but orthoses were worn in both shoes.

### Procedures

All participants were initially screened based on measures of arch height index (AHI) and were only included if they fell within the normative range reported by Butler et al. [18]. These authors [18] reported that the mean AHI for a group of recreational runners was 0.363 ± 0.030 for sitting and 0.340 ± 0.030 for standing and the AHI between genders was similar. Thus, the AHI values for the 20 participants fell within these values for both sitting and standing.

**Figure 2:**
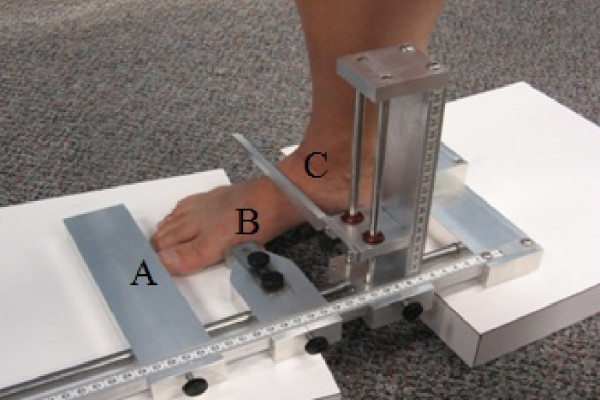
**Arch Height Index Measurement System**. Adjustable sliders were used to measure total foot length (A), truncated foot length (B), and dorsal height at 50% of total foot length (C).

AHI was measured using a custom built Arch Height Index Measurement System [18]. Two boards were placed under the foot, one under the calcaneus and one under the forefoot to allow the midfoot to achieve maximum deformation (Figure 2). The measure of AHI is unitless and was defined as the ratio of dorsum height at 50% of total foot length, divided by the foot length from the back of the heel to the head of the first metatarsal, defined as the truncated foot length [19]. Seated AHI was obtained with the participant seated, with hips and knees flexed to 90 degrees, and approximately 10% of total body weight on the foot. Standing AHI was obtained with the participant standing with equal weight on both feet. The AHI measurement was deemed an appropriate measurement of static foot structure as its very good to excellent reliability has been previously demonstrated in the literature [18,19].

**Figure 3:**
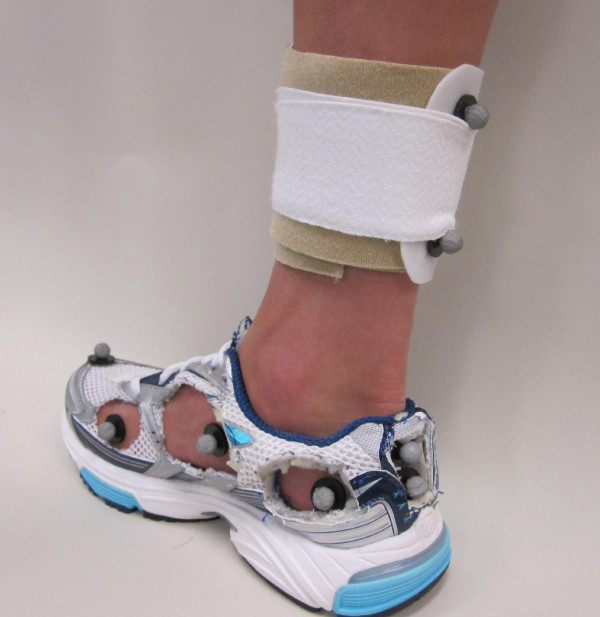
**Marker set-up for kinematic data collection**.

Three-dimensional treadmill walking data were collected using an eight-camera motion analysis system (Vicon Motion Systems Ltd, Oxford, UK). All participants were barefoot and fitted with 9 mm retroreflective markers adhered directly to the skin on various anatomical landmarks of the tibia, fibula and foot (Figure 3). Specifically, a hard plastic shell with four markers was placed on the lower one-third of the tibia/fibula to represent the shank segment. The rearfoot segment was defined using a cluster of three tracking markers with two markers placed superior and inferior along the long axis of the calcaneus (SCAL, ICAL) and one placed near the sustentaculum tali on the medial aspect of the calcaneus (MCAL). Additional tracking markers were placed on the navicular tuberosity (NAV), distal aspect (head) of the first metatarsal (D1MT), and distal and superior aspect of the shoe. Two additional anatomical markers were placed on the lateral and medial malleoli to represent the ankle joint and establish the local joint coordinate system.

Specific holes were cut in the neutral laboratory running shoes (Brooks Glycerin) to allow the tracking markers to be recorded by the cameras and measure rearfoot and midfoot kinematics. Kinematic data were collected for three shod walking conditions (no-orthotic, moulded orthotic, non-moulded orthotic) and the order of condition was randomized amongst the participants. The non-moulded condition consisted of simply removing the semi-custom orthoses (SCO) from the packaging and placing them in the shoe. The moulded condition was consistent with the manufacturers recommended heat-moulding procedures for the SCO and the no-orthotic condition had only the shoe manufacturer's liner placed within the shoe.

Between the three conditions, the D1MT, MCAL and NAV markers were removed from the foot while the orthotic condition was changed. To ensure near-identical marker placement for each walking trial, a circle the size of the marker base, was stamped on the foot and the marker was placed in the centre of this circle for each trial.

A standing calibration of 1 second was obtained with the participant's feet placed 0.30 m apart and pointing directly forward and orthogonal to the global laboratory coordinate system. Following the standing calibration, the participants were provided a one-minute warm-up period to walk on the treadmill at 1.2 ms^-1^. Following the familiarization period, marker trajectory data were captured at a rate of 120 Hz.

Ten continuous footfalls of the treadmill walking trial were selected for analysis. Raw marker trajectory data were filtered using a fourth-order low-pass Butterworth filter at 12 Hz. Anatomical coordinate systems were created for the shank and rearfoot segments using Visual 3D software (C-motion Inc, Rockville, USA). Only the stance phase of gait was analysed and all kinematic data and raw marker trajectories were normalized to 101 data points prior to data processing. Stance phase was defined as initial heel contact to toe off using a kinematic velocity-based algorithm [20] applied to the SCAL marker and toebox marker, respectively.

### Data processing

**Figure 4:**
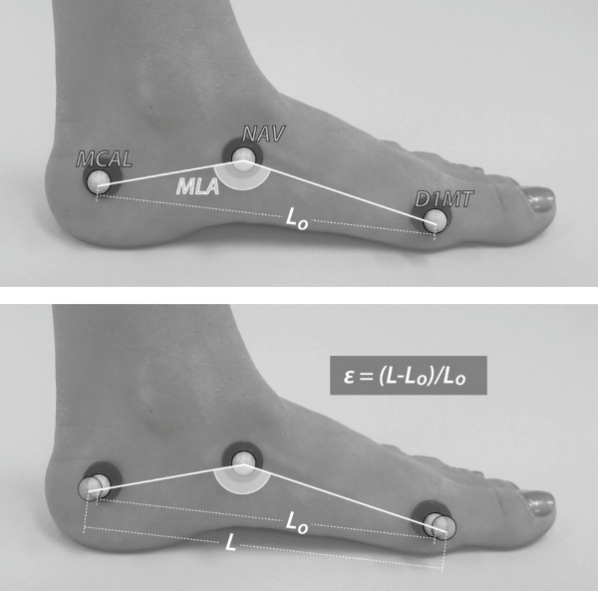
**Representation of how MLA angle and PFS values were calculated using the retroreflective markers**. Top: calculation of medial longitudinal arch (MLA) angle. Bottom: calculation of plantar fascia strain (PFS or ε) as the change in marker position.

Cardan angles were used to calculate three-dimensional angles for the rearfoot and shank. Rearfoot eversion was expressed as frontal plane motion relative the shank segment. Raw marker trajectories in the global coordinate system were exported for the D1MT, NAV, and MCAL markers for the purpose of calculating plantar fascia strain (PFS) and medial longitudinal arch (MLA) angle values. The MLA angle was calculated in a manner similar to Tome et al. [21]. The MLA angle was defined as the angle subtended by two lines, one from the marker on the medial aspect of the calcaneus (MCAL) to the navicular tuberosity (NAV) and the other from the head of the first metatarsal (D1MT) to the NAV marker (Figure 4). Plantar fascia strain is a unitless measure calculated by approximating the plantar fascia as spanning between the first metatarsal head (D1MT) and medial calcaneus marker (MCAL) and determined as change in relative marker position.

We had previously reported root mean squared error of 1.1 degrees for changes in measures of forefoot sagittal plane angles as a result of removing the D1MT marker [22]. However, we conducted a separate experiment to determine the between-condition measurement error for PFS caused by removing and then placing the markers back within the stamped circles. On a separate occasion, three of the participants returned to the laboratory and had the same circles stamped on their foot, the shoe placed on their foot, and the D1MT, NAV, and MCAL markers placed within the circles. Walking gait kinematic data were collected in the same manner described for the no-orthotic condition. The markers and shoe were then removed, placed back on, kinematic data were collected once again, and this procedure was then repeated a third time. Subsequent calculations of PFS and MLA angle were made for the three separate data collections.

Custom Labview software (National Instruments Corp, Austin, USA) was used to calculate discrete kinematic variables of interest. These variables included 1) peak rearfoot eversion, 2) peak tibial internal rotation, 3) peak MLA angle and 4) peak PFS.

### Data analysis

Between-condition statistical comparisons were made using repeated measures analysis of variance (ANOVA) for the variables of interest with an alpha level of *p *< 0.05. Bonferroni post-hoc tests were used to determine differences, if any, between the three conditions (*p *< 0.05). With three conditions, and thus two degrees of freedom, a priori comparisons were planned between 1) SCO moulded and SCO non-moulded and between 2) SCO moulded and no-orthotic conditions. Finally, we calculated Cohen's d effect sizes to better understand potential differences, if any, between orthoses conditions.

For the separate between-condition measurement error experiment, descriptive statistics were calculated for the average change in distance between the D1MT and MCAL markers. All analyses were undertaken using SPSS 17.0 (SPSS Inc, Chicago, USA).

## Results

### Measurement error study

The average change in distance between the D1MT and MCAL markers was 0.15 mm (± 0.01) with the average percent change in PFS equal to 14.46% (± 5.38).

### Between condition study

**Figure 5:**
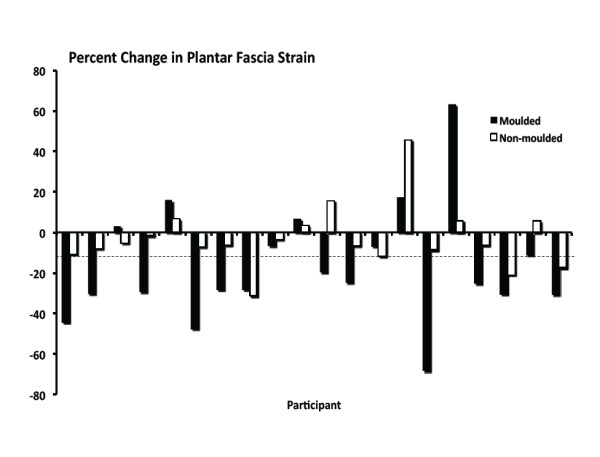
**Change in plantar fascia strain for each of the twenty participants while walking in the moulded and non-moulded conditions**. Negative values indicate a reduction in strain as compared to the no-orthotic condition. The dashed line approximates the 14% measurement error.

**Figure 6:**
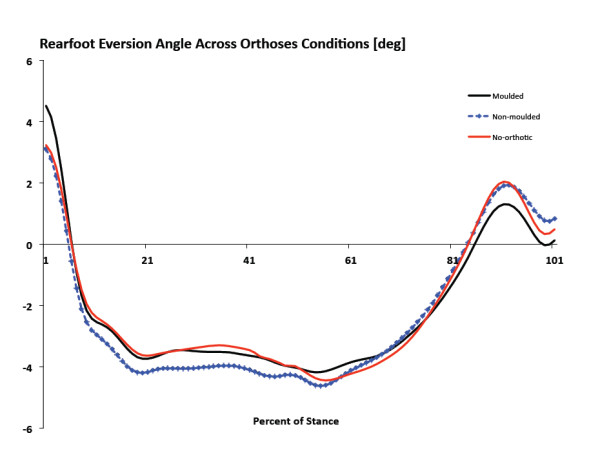
**Ensemble mean average kinematics curves for frontal plane rearfoot motion**. Positive values indicate rearfoot inversion and negative values indicate eversion.

**Figure 7:**
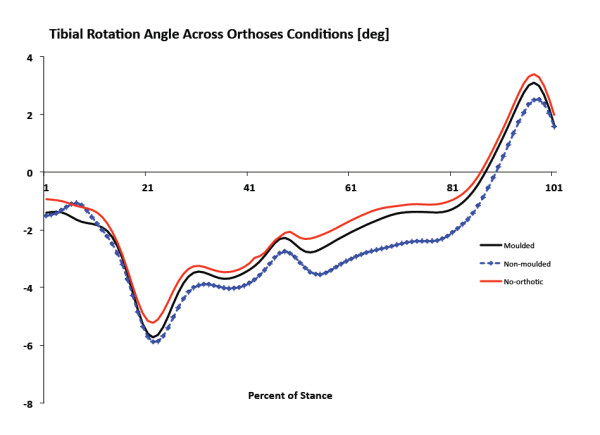
**Ensemble mean average kinematics curves for transverse plane shank motion**. Positive values indicate tibial external rotation and negative values indicate internal rotation.

**Figure 8:**
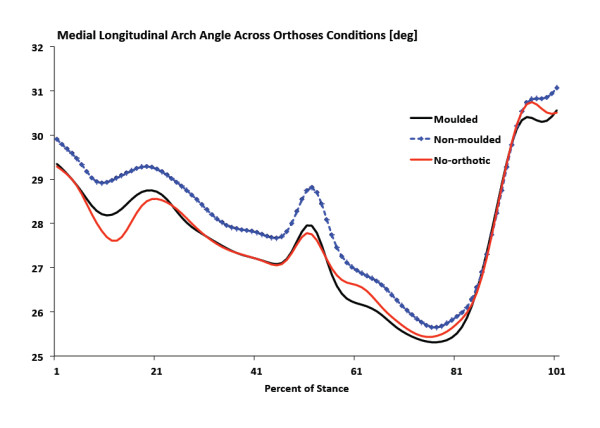
**Ensemble mean average kinematics curves for medial longitudinal arch angle**. Values closer to zero indicate arch deformation.

A summary of between-orthoses changes in the variables of interest is provided in Table 1 and Figures 5, 6, 7 &8. As can be seen in Figure 5, PFS was significantly reduced for SCO moulded compared to the no-orthotic condition. Specifically, an average 34.77% reduction in PFS was measured between the SCO moulded and no-orthotic conditions for all participants. Twelve of the twenty participants exhibited greater than a 14.46% decrease in strain while walking in the SCO moulded condition while two others exhibited a 6% and 12% decrease. No differences were measured between SCO moulded and SCO non-moulded conditions but thirteen of the twenty participants also showed decreases in PFS while walking in the moulded orthotic condition as compared to the non-moulded.

**Table 1:** Summary of the variables of interest (Mean, (SD)) for plantar fascia strain (PFS), medial longitudinal arch (MLA) angle, peak rearfoot eversion (RFEv) angle, and peak tibial internal rotation (TibRot) angle across the three orthoses conditions

Variable	No-orthotic	Condition Moulded	Non-moulded
PFS	0.08 (0.01)	0.05 (0.02) **ES = 0.71; p = 0.03**	0.06 (0.02) ES = 0.44; p = 0.10
MLA	25.40 (8.07)	25.30 (7.72) ES = 0.03; p = 0.48	25.62 (6.87) ES = 0.07; p = 0.45
RFEv	4.44 (4.58)	4.18 (1.60) ES = 0.21; p = 0.34	4.63 (1.51) ES = 0.03; p = 0.49
TibRot	-5.23 (1.47)	-5.75 (2.33) ES = 0.28; p = 0.20	-5.89 (2.14) ES = 0.07; p = 0.44

Note: Effect sizes (ES) and p-values under Moulded are in comparison to No-orthotic whilst ES and p-values values under Non-Moulded are in comparison to Moulded.

There were no significant differences in peak RFEV between no-orthotic and SCO moulded or between SCO moulded and SCO non-moulded (Figure 6). No significant differences were measured for peak tibial internal rotation between no-orthotic and SCO moulded or between SCO moulded and SCO non-moulded (Figure 7). Finally, there were no significant differences for peak MLA angle between no-orthotic and SCO moulded or between SCO moulded and SCO non-moulded (Figure 8).

## Discussion

The purpose of this study was to determine changes in multi-segment foot biomechanics during shod walking with and without an orthotic device. To our knowledge, this is the first study to investigate the effect of orthoses on midfoot kinematics to better understand their clinical efficacy.

In support of the hypotheses, the results of the present study indicate that semi-custom orthoses reduce plantar fascia strain compared to walking without an orthoses. While we are not aware of another gait study that has calculated strain within the plantar fascia, the results are consistent with previous hypotheses of how orthoses may function to treat plantar fasciitis and minimize arch deformation [16,17,23]. Moreover, the majority of participants exhibited a larger than 14% reduction in strain, the between-condition measurement error. Finally, the effect size was large for the comparisons suggesting that the measure of strain has biological significance.

The results of the present study are supported by Kogler et al. [24] who conducted a cadaveric study and surgically implanted a strain transducer in the plantar aponeurosis. Measurements of plantar fascia strain during five orthoses conditions were recorded whilst axial loads were applied to the tibia to simulate weight bearing. These authors reported that only three of the five orthoses significantly reduce strain in the plantar fascia suggesting that certain types of orthoses are more effective than others in supporting the longitudinal arch. Specifically, the pre-fabricated orthoses and one of the custom orthoses did not reduce strain whereas the three custom-made orthoses significantly reduced strain across axial loading conditions. While the orthosis used in the current study was considered a semi-custom device, it too reduced PFS. How this type of device compares to custom-made orthoses is unknown but future research is necessary.

It is interesting to note that as early as 2003, Williams et al. [15] stated that orthotic devices may provide more control of the midfoot than the rearfoot but no study has since undergone this type of investigation. These authors [15] also went on to state it is likely that evaluation of the midfoot may provide more complete information regarding the exact control and efficacy of orthotic devices. To our knowledge, this is the first study to report on changes in plantar fascia strain when walking in an orthotic device. Indeed, the use of an orthotic device has been recommended as the primary method for the treatment of plantar fasciitis [23-26]. Therefore, the results of the current study suggest that the treatment of foot and ankle injuries such as plantar fasciitis may be due, in part, to reductions in plantar fascia tissue strain. Future research involving custom-made orthotic devices are necessary especially in light of the non-significant findings of other kinematic variables of interest.

No differences in MLA angle were found between conditions. While previous studies have reported that MLA angle differs between individuals with mid-stage posterior tibial tendon dysfunction (PTTD) and controls [21] other studies involving early-stage PTTD [27] have shown no differences. Moreover, Tome et al. [21] measured the difference between standing MLA angle, normalized to subtalar neutral position, as compared to the peak MLA angle. Since we did not obtain MLA angle values in a subtalar neutral position, we are not able to directly compare our results to those of Tome et al. [21]. In addition, the present study was limited in that the vertical height of the medial calcaneal marker from the plantar surface was not standardized. However, the within-subject comparisons of the present study would make this a moot point. Regardless, future studies that carefully standardize the marker placement are required to confirm or rebuke whether MLA angle is a measure best suited to healthy participants or whether it is for more pathological patients such as mid- or late-stage PTTD.

Contrary to the hypotheses, there were no differences in average peak rearfoot eversion or tibial internal rotation angles across the three conditions. Since there is no external medial posting material on the heel counter of the semi-custom orthotic device used in the current study, the similar peak rearfoot eversion and tibial internal rotation values between conditions are not completely unexpected. While the effect of orthoses on rearfoot kinematics has been well documented [1-6], and since foot orthoses are typically designed to control rearfoot eversion, we hypothesized they would reduce the relative amount of eversion to tibial internal rotation motion [28,29].

A number of studies [30-32] have assessed the effect of foot orthoses on tibial (shank) motion reporting decreases of 2-4 degrees in peak tibial internal rotation and internal tibial rotation excursion. Nawoczenski et al. [31] studied the effects of semi-rigid posted orthoses on three-dimensional lower leg kinematics and found no significant change in foot eversion. However, mean internal tibial rotation was reduced by 2 degrees compared to not using orthoses. Eng and Pierrynowski [32] reported that rearfoot eversion was decreased by 1-3 degrees and internal tibial rotation was reduced by 0.5-2 degrees when using foot orthoses. Unfortunately, these studies utilized a custom-made orthotic device so comparisons to the current study are difficult.

To our knowledge, only two studies have investigated biomechanical differences between custom and semi-custom orthoses [33,34]. Overall, both studies reported that there were little to no differences in rearfoot kinematics between the two different devices while running or walking. Pfeffer et al. [23] conducted a prospective, randomized, single-blinded clinical trial and reported that when used in conjunction with a stretching programme, an over-the-counter prefabricated foot orthoses is more likely to reduce symptoms associated with plantar fasciitis compared to custom-made orthoses. Moreover, Landorf et al. [35] investigated the effectiveness of different foot orthoses in the treatment of plantar fasciitis. These authors reported that after three months of treatment, reductions in pain and improvements in function were measured only for the over-the-counter prefabricated and customized orthoses as compared to sham orthoses. Thus, an over-the-counter orthotic device appears to function in a manner comparable to a custom-made device. However, the current study did not compare the over-the-counter semi-custom device to a custom-made orthotic and future research is necessary.

We chose to investigate a semi-custom orthotic device that incorporates a heat-moulding process, to further understand if the moulding process would significantly alter rearfoot or midfoot kinematics and plantar fascia strain as compared to a no-orthotic condition. We hypothesized the semi-custom device, whether moulded or non-moulded, would reduce peak rearfoot eversion, peak tibial internal rotation, and medial longitudinal arch angle, compared to the no-orthoses condition. However, no differences were found between orthoses conditions.

We hypothesized that the non-moulded orthotic condition would serve to minimize arch deformation, and thus reduce plantar fascia strain and medial longitudinal arch angle, more so as compared to the moulded condition as a direct result of the heat-moulding process and material deformation. Again no differences were found between orthoses conditions suggesting that heat moulding does not change rearfoot or midfoot kinematics. However, inspection of Figure 5 shows that for 13 of the 20 participants, a greater reduction in plantar fascia strain occurred when walking in the moulded condition as compared to the non-moulded and the average reduction in strain between conditions was 24.62% (± 14.16). Irrespective of the fact that the moulded condition resulted in overall greater reductions in PFS compared to the non-moulded condition, perhaps the large variability accounted for the lack of significant differences between conditions. Moreover, perhaps the heat-moulding process and material deformation according to the shape of the individual's arch is ideal to reduce tissue strain and optimize the orthotic device, which is contrary to the original hypothesis. Future research involving a larger sample size and involving individuals with differing foot structural characteristics is necessary to answer these questions.

Several limitations are acknowledged. First, the present investigation was limited by the fact that the AHI was the only structural measurement of the foot. For instance, the range of motion of the rearfoot might influence the degree to which an individual's rearfoot eversion can change when walking in an orthotic device. One could also question whether a change in arch height would be expected during walking gait from individuals with a typical AHI value. Ideally, future research involving excessively mobile feet, based on AHI criteria, in comparison to the healthy participants involved in the present study are necessary to better understand the role of orthoses in reducing PFS and MLA angle. Second, the current results are only applicable to walking and cannot be extrapolated to running. Since many chronic injuries, such as plantar fasciitis, occur in response to atypical loading during running, future running-related research is necessary to understand how an orthoses might affect strain. Third, the examiner responsible for data collection and analysis of the data was not blinded to orthoses condition. However, we randomized the order of conditions, coded the trials (T1, T2, T3) the same for all participants, and only after data processing revealed the order of conditions. Fourth, the plantar fascia runs from the calcaneal tuberosity to the heads of the first through fifth metatarsal bones [36] and encounters tensile and torsional stress as components of normal physiological function [37]. We modelled the tissue and approximated its location from the medial aspect of the calcaneus to the head of the first metatarsal, which is a simplified representation. Future research involving finite element modelling [37] and/or incorporation of such equipment as real-time fluoroscopy, in parallel with motion capture, may be better suited to provide more accurate measures of tissue strain. For example, Wearing et al. [38] used digital fluoroscopy and concluded that compared to controls, arch shape and arch angle were similar but plantar fascia thickness was greater for participants experiencing chronic plantar fasciitis. Such research may help to understand the role of orthoses and help optimize treatment options for injured patients. Finally, since the change in position of the D1MT and MCAL markers were used to calculate the PFS, and since PFS was significant (indicating a change in marker position), one must assume that the only reason the MLA angle was not significantly different amongst the three conditions studied was lack of movement of the NAV marker. Using 2-D roentgen photogrammetry, Tranberg and Karlson [39] reported that in relation to the underlying bones, the navicular marker moved up to 1.97 mm in the superior-inferior direction. Thus, and as previously discussed, future research using such technology as real-time fluoroscopy, in parallel with motion capture, is necessary.

## Conclusions

This is the first study to investigate the effect of an orthosis on midfoot biomechanics. Our findings indicate that semi-custom moulded orthoses reduce plantar fascia strain compared to walking without an orthoses. However, this particular device does not control peak rearfoot eversion, tibial internal rotation, or arch deformation. Heat-moulding the orthotic device does not have a measurable effect on the biomechanical variables compared to the non-moulded condition.

## Competing interests

The authors declare that they have no competing interests.

## Authors' contributions

RF and BB developed the rationale for the study, designed the study protocol, conducted the data collections, processed the data, and drafted the manuscript. All authors have read and approved the final manuscript.
